# Antithrombotic strategy in cancer patients comorbid with acute coronary syndrome and atrial fibrillation

**DOI:** 10.3389/fcvm.2023.1325488

**Published:** 2023-12-12

**Authors:** Tianbo Wang, Xiaohan Liu, Yuxin Zhu, Yue Zhang, Zhen Zhang, Gang Huang, Junbo Xu

**Affiliations:** ^1^ Department of Cardiology, The Third People’s Hospital of Chengdu, Chengdu, China; ^2^ Affiliated Hospital of Southwest Jiaotong University, College of Medicine, Southwest Jiaotong University, Chengdu, China; ^3^ Cardiovascular Disease Research Institute of Chengdu, Chengdu, China

**Keywords:** cancer, atrial fibrillation, acute coronary syndrome, coronary artery disease, antithrombotic therapy

## Abstract

It has been shown that patients with cancer have a longer expected life duration, benefiting from advanced medical therapy. Meanwhile, the risk of suffering from cardiovascular disease (CVD) has been increasing with ageing. A growing number of studies have elucidated the association between cancer and CVD. Cancer, atrial fibrillation (AF) and coronary artery disease share some common factors and interact with each other, such as obesity, aging, diabetes, and inflammation, but the potential specific mechanism is still unclear. In addition, cancer-specific and therapy-related factors may increase the risk of embolism and bleeding in patients with cancer than in general population. However, current available embolic and bleeding risk scores applied in patients with CVD may not be applicable for risk assessment in cancer patients, which would be difficult for clinicians to select an appropriate antithrombotic regimen and ensure the balance between bleeding and embolism. Moreover, different types of cancer have distinct risks, which may increase the complexity of antithrombotic therapy. In this review, we review the literature related to cancer, AF, and acute coronary syndrome, focusing on the epidemiological status, physiological mechanism, embolism and bleeding risks, and strategies of antithrombotic therapy.

## Introduction

Cardiovascular disease and cancer have been considered to be the two leading causes of death in developed countries (1, 2). For decades, with continuous advances in cancer screening, diagnosis and therapies, the number of cancer patients and survivors has increased steadily (2, 3). Due to the increase of life expectancy in cancer patients and the impact of cancer therapies, the risk of cancer patients complicated with coronary arterial disease (CAD) and AF is also increasing. Although risk assessment of embolism and bleeding in patients with AF and ACS is relatively perfect, including embolism and bleeding risk scores (CHA_2_DS_2_-VASc score and HAS-BLED score) and relevant guidelines (4, 5), the factor of cancer is not included in these scores. Cancer-specific and therapy-related risk factors may lead to an increased risk of embolism. Moreover, patients with cancer have a higher risk of bleeding than those without cancer (6, 7). Thus, the balance of thrombotic and bleeding risks has become a thorny issue in the antithrombotic therapy of cancer patients with AF and acute coronary syndrome (ACS). Additionally, most cardiovascular randomized controlled trials exclude cancer patients, and there are few relevant guidelines to guide antithrombotic therapy, which is a gap in clinical practice. Therefore, this paper reviews the prevalence, potential pathological mechanism and interaction of AF and ACS and current evidence of antithrombotic therapy in patients with cancer.

## Epidemiology

Since the 1990s, cancer-related mortality has steadily declined, which has led to a steady increase of cancer survivors (2, 8, 9). As the second leading cause of death after CVD, cancer has been the leading cause of death in high-income countries (1). In 2020, it is estimated that 19.3 million new cancer cases were diagnosed, in which breast cancer was become the most common diagnosed cancer (11.7%) compared with lung cancer (11.4%), followed by colorectal cancer (10.0%), prostate cancer (7.3%) and gastric cancer (5.6%) (2).

AF is the most common cardiac arrhythmia and affected nearly 59,700 thousand people worldwide until 2019 (9). The lifetime risk of AF is estimated to be one in four in both men and women at age 40 years and older (10). Patients with cancer may have a higher risk of AF than those without cancer. In a prospective study including 1,045 patients (11), the incidence of AF in cancer patients was higher than that in noncancer patients during the mean follow-up time of 16.3 years (HR 2.47; 95%CI: 1.57–3.88). Recently, an American nationwide epidemiological study on 85,423 patients with breast cancer showed that 9,425 patients had AF prior to the diagnosis of breast cancer, and 2,993 patients had new-onset AF within one year after the diagnosis of breast cancer [incidence 3.3%, 95% CI: 3.0%–3.5%, at 1 year; higher rate in the first 60 days (0.6%/month)] (12). ACS is regarded as a cardiovascular complication that requires special attention in cancer patients. Data from the national inpatient sample (NIS) database of 6,563,255 patients with acute myocardial infarction (AMI) between 2004 and 2014 showed that the incidence of cancer was 9%. Among them, prostate cancer, breast cancer, colon cancer and lung cancer are most closely linked with AMI (13). In a prospective, multicenter special project university medical ACS cohort, the incidence of cancer was 7.74% in 2,132 ACS patients (14).

The epidemiological data are relatively limited in cancer patients with AF and ACS. In the analysis of the ENGAGE AF-TIMI 48 Trial (15), a significant relationship between malignancy and CAD was observed. (*p* = 0.017). In line with this, the baseline of the ORBIT-AF registry shows that the incidence of prior myocardial infarction in cancer patients is 2.7% higher than that in noncancer patients (*p* = 0.02) (16). In another observational study (17), a difference was found in the prevalence of ACS in AF patients with and without cancer (*p* < 0.001). In short, it is undeniable that cancer patients have a higher risk of CVD than noncancer patients (18).

## Pathophysiology in cancer, AF and ACS

### Cross talk between cancer and platelets

Cancer and platelets can interact with each other, which can be simply understood as tumor cell induced platelet activation and aggregation and activated platelets participate in every step of cancer development by promoting tumor growth, angiogenesis, metastasis, and cancer-related thrombosis (19). Circulating tumor cells (CTC) can induce platelet activation and aggregation (tumor cell induced platelet aggregation, TCIPA), leading to tumor thrombi formation (20, 21). In addition, the association between tumor cells and platelet activation and aggregation may also be related to tissue factors (19, 21). In fact, as early as the 19th century, studies have reported a correlation between thrombocytosis and poor prognosis in cancer patients (22). Platelets can promote tumor angiogenesis and vascular remodeling. Platelets contain various angiogenic factors, which can affect angiogenesis and indirectly promote angiogenesis, such as vascular endothelial growth factor (VEGF), fibroblast growth factor, platelet-derived growth factor (PDGF), insulin-like growth factors (IGF-I), tissue growth factor β (TGF-β), platelet factor 4 (PF4) and so on (23). However, studies have found that PF4 has an inhibitory effect on tumor angiogenesis (24). Platelets also play an important role in the invasion and metastasis of tumor cells. Activated platelets can encapsulate CTC through integrin, fibrin, and P-selectin (25, 26). Subsequently, activated platelets secrete adherents and bind to the surface of CTC, protecting them from shear stress and immune cell attacks. In addition, the adhesion molecules expressed by platelets can also help CTC form active and persistent adhesion with the endothelium, promoting extravasation and seeding of metastatic lesions (27).

### Cancer and thrombosis

The increased risk of thrombosis in cancer patients may be explained by Virchow's triad, including blood stasis, endothelial injury or vessel walls injury, and hypercoagulability. In cancer patients, each of the three components Virchow's triad that are prone to thrombosis have abnormalities, thus meeting the requirements for prethrombosis or hypercoagulability (28). Mechanical compression and inactivity of tumors may be potential factors which lead to blood stasis and thromboembolism. Long bed rest after cancer treatment surgery is associated with venous thromboembolism (29). The use of anticancer drugs, inflammation and the production of neutrophil extracellular traps may be associated with endothelial damage and activation in cancer patients (30). In addition, potential mechanisms of hypercoagulability in cancer patients may include expression of tissue factors, release of prethrombotic substances, and upregulation of heparinase (30, 31).

### Pathogenesis of AF and cancer

The potential mechanism between AF and cancer may be explained by the following assumptions: (1) Cancer-related systemic inflammation may lead to electrical and structural remodeling of the atrium, which causes AF. AF may induce new inflammation, leading to new AF-the so-called “AF causes AF” (32–35). (2) Some factors of cancer, including pain, infection, metabolic abnormalities, emotion, and physical pressure, may cause an imbalance in the autonomic nervous system, which causes AF (36). (3) The occurrence of AF is also associated with cancer therapies that may be linked to mitochondrial dysfunction, oxidative stress, inflammation, left ventricular dysfunction and necrosis (37). A study showed that doxorubicin can induce Ca/calmodulin-dependent protein kinase II (CaMKII)-mediated Ca^2+^ leakage in the sarcoplasmic reticulum to disrupt intracellular Ca^2+^ homeostasis. Increased sarcoplasmic reticulum calcium leakage is also a potential mechanism for inducing AF (38). In addition, the risk of AF may be increased by ibrutinib (39, 40), potentially through inhibition of cardiac PI3K-AKT signaling and production of reactive oxygen species (ROS).

### Anticancer therapy-related coronary artery disease

Cancer therapy-related modalities (e.g., chemotherapy, radiotherapy, immunosuppressive therapy, and targeted therapy) are associated with an increased risk of CAD.

Platinum may induce cardiotoxicity due to endothelial injury, platelet aggregation, and thrombosis (41, 42). The mechanism of cardiotoxicity induced by fluorouracil is unknown. However, coronary thrombosis, coronary vasospasm, vascular endothelial injury, direct cardiotoxicity, and oxidative stress have been proposed (43–45).

Targeted drugs may increase the risk of CAD by damaging the vascular endothelium, arterial thrombosis, and vasospasm (46). Vascular endothelial growth factor (VEGF) can promote cell proliferation, endothelial cell migration and angiogenesis. Some anti-VEGF drugs, such as bevacizumab, sorafenib, and sunitinib, may reduce endothelial cell function, contributing to the increased risk of endothelial cell injury and thrombotic events (47). Sorafenib can also cause CAD by inducing vasospasm (48).

Radiotherapy may cause vascular endothelial damage by activating lysosomal enzymes in the intima and media of blood vessels, which can promote the formation of cholesterol plaques after a few days of radiotherapy (49, 50). In addition, radiotherapy may also cause fibrosis of the intima, media, and adventitia of blood vessels, thus accelerating the development of arteriosclerosis (51, 52).

### The potential common mechanism among cancer, AF and CAD

Cancer, AF and CAD share common risk factors (53), including smoking, obesity, diabetes, hypertension and lack of exercise. Obesity is regarded as the main risk factor for CVD (54). A study showed that 20% of all cancer patients may be related to weight gain and obesity (55). Insulin resistance, dyslipidemia and inflammation commonly occur in obese patients and can lead to CVD and cancer (56–59). Diabetes not only increases the risk of CVD by 2–5-fold (60) but can also increase the risk of cancer, especially colorectal cancer (61). Diabetes mellitus may induce cancer through a variety of factors, including excessive reactive oxygen species (ROS) formation, oxidative stress, chronic inflammation, and mitochondrial dysfunction (62). Smoking is closely related to the development of CVD. Tobacco ingredients, including carbon monoxide and oxidants, may increase oxidative stress and reduce the level of nitric oxide that causes endothelial damage, inflammation, and insulin resistance to affect atherosclerosis (63–65). Smoking can also induce cancer through ROS and oxidative damage (66).

Inflammation exists in many diseases, including CVD and cancer (67–69). In 1863, Virchow found white blood cells in tumor tissue and proposed the hypothesis that cancer may originate from chronic inflammation (70). Cancer, AF, and CAD may be linked to inflammation (32, 67, 68). The NLRP3 (Nacht, LRR, and PYD domain containing protein 3) inflammasome can be activated by AF and mediate the release of IL-1B, which leads to the fibrosis of cardiomyocytes, accelerating atrial remodeling and causing the generation of new AF (71, 72). Animal models have also demonstrated that the NLRP3 inflammasome and IL-1 promote arteriosclerosis and increase arterial thrombosis (73–75). IL-1 can promote tumor proliferation and increase the invasiveness of cancer cells in chronic inflammation. IL-6 is secreted by activated monocyte macrophages. In the atrium, there was a significant positive correlation between IL-6 levels and extracellular matrix volume in patients with AF (76). The increase in the interstitial extracellular matrix is associated with atrial remodeling, which may maintain AF (77). Meanwhile, IL-6 has also been confirmed to be associated with vascular endothelial injury and atherosclerosis (78, 79).

## Risk of embolism and bleeding in cancer patients comorbid with AF and ACS

### Thromboembolism

Cancer was not included in the embolism risk score, including the CHA_2_DS_2_-VASc score (80) (congestive heart failure, hypertension, 75 years old or older, diabetes, stroke/transient ischemic attack/thromboembolism, vascular disease, 65–74 years old, sex) and ATRIA score (81) (previous ischemic stroke, age, female, diabetes, congestive heart failure, hypertension, proteinuria, eGFR < 45 or ESRD). A study comparing the usefulness of the CHADS_2_ or CHA_2_DS_2_-VASc score in AF patients diagnosed with cancer showed that the CHADS_2_ score is more predictive of increased stroke risk in AF patients with cancer than the CHA_2_DS_2_-VASc score, which is also the largest study to analyze stroke risk in AF patients with cancer (82).

The risk of embolism in AF patients with cancer is not certain. On the one hand, cancer may not increase the risk of embolism in AF patients. Data from the ARISTOTLE trial (83) showed that no significant difference was observed in the risk of stroke/systemic embolism (SE) in AF patients with or without cancer, however, the superior efficacy and safety of apixaban vs. warfarin were consistent. A similar result was obtained from the ROCKET-AF trial: it seemed that cancer status had little association with stroke in AF patients (76). However, cancer increases the risk of embolism in AF patients. In a prospective study with nonvalvular AF patients (84), the incidence of thromboembolic events (ischemic stroke/transient ischemic attack and SE) in cancer patients was higher than that in noncancer patients (adjusted HR 2.58, 95% CI 1.08–6.16, *p* = 0.033). Similar to a French observational, retrospective cohort study including 2,435,541 AF patients (85), pancreatic cancer and breast cancer patients had a higher risk of thromboembolism than noncancer patients [IRR (1.2; 95%CI, 1.0–1.4) *p* = 0.02; (1.1; 95%CI, 1.0–1.2) *p* = 0.0002, respectively].

Cancer patients are more likely to have a higher risk of recurrent myocardial infarction after percutaneous coronary intervention (PCI). Data from the US National readmission database showed that the 90-day readmission rate for acute myocardial infarction (AMI) after PCI was significantly increased in active cancer (86), including nonmetastatic cancer (OR 1.28, 95%CI: 1.20–1.37, *p* < 0.001) and metastatic cancer (OR 1.63, 95%CI: 1.38–1.93, *p* < 0.001). In the subgroup analysis, AMI readmission rates within 90 days were higher in patients with active cancer (12.1% for lung cancer, 10.8% for colon cancer, 7.5% for breast cancer, 7.0% for prostate cancer, and 9.1% for all cancers) than in patients without cancer (5.6%) (*p* < 0.05). Malignancy was the strongest predictor of stent thrombosis in ACS patients undergoing PCI (87). Another study exploring the risk of thromboembolism after PCI in cancer patients also showed that during the 5-year follow-up period (88), cancer patients had a higher incidence of MI after PCI than noncancer patients (16.1% vs. 8.0%; HR 2.10; 95% CI: 1.49–2.96; *p* < 0.001). Other scores for evaluating ACS prognosis also do not include cancer (89).

It is not self-evident that the risk of embolism in AF patients with cancer may be different from what we imagine. The risk of thromboembolism may be higher in specific cancers (e.g., lung cancer, pancreatic cancer, breast cancer). In addition, although our commonly used AF score does not include cancer, it still has some predictive power.

### Bleeding

Cancer is also not included in the common bleeding scores, including the HAS-BLED score (90), ORBIT (91) and ATRIA (81) bleeding risk score. Although cancer is considered an independent predictor of major bleeding in the complete ORBIT bleeding model, cancer is not included in the final 5-factor score (91). An observational retrospective cohort study, including 399,344 patients with AF and cancer comparing the role of HAS-BLED, ORBIT and ATRIA bleeding risk scores, showed that three scores were significantly linked to major bleeding, GI bleeding, and intracranial hemorrhage (ICH) (92). Moreover, the HAS-BLED score performed better than other scores in ICH prediction, while the ORBIT score showed the best prediction for major bleeding and GI bleeding (*p* < 0.001 for all AUC comparisons) (92). In a Swedish national study to validate the PRECISE-DAPT score, the PRECISE-DAPT score could predict major bleeding in the cancer group but with only poor or moderate discriminative capability (c-statistic 0.59; 95%CI, 0.53–0.66) (93). A retrospective registry-based cohort study of Spain including 1,137 patients with AF and cancer compared the predictive efficacy of HAS-BLED score, ATRIA score, and HEMORR2HAGES score on bleeding risk. The result showed that all scores were poor in patients with cancer (c-statistic <0.6 and Brier score >0.1). HAS-BLED score (c-statistic 0.56 and Brier score 0.17) has better predictive performance, compared to ATRIA score (c-statistic 0.55 and Brier score 0.17) and HEMORR2HAGES score (c-statistic 0.54 and Brier score 0.17) (94). Available bleeding risk scores are shown in Table 1.

**Table 1:** Current available bleeding risk scores for patients with cardiovascular disease under antithrombotic therapy.

Score	Risk factors	Ref
HAS-BLED	Hypertension, Liver and kidney function, Stroke, Bleeding, INR, Age, Drug/Alcohol	(90)
ORBIT	Antiplatelet therapy, Renal function, Presence of anemia/abnormal hemoglobin, Bleeding, Age	(91)
ATRIA	Previous ischemic stroke, Female, Diabetes, CHF, Hypertension, Age, Proteinuria, ESRD or eGFR < 45ml/min/1.73m2	(81)
HEMORR2HAGES	Hepatic or Renal disease, Ethanol abuse, Malignancy, Older (age >75 years), Reduced platelet count or function, Rebleeding risk, Hypertension (uncontrolled), Anemia, Genetic factors (CYP2C9 single nucleotide polymorphisms), Excessive fall risk (including neuropsychiatric disease), and Stroke.	(94)
PARIS	Age, BMI, Triple therapy at discharge, Anemia, Current smoking. Renal dysfunction	(95)
PRECISE-DAPT	Age, Creatinine clearance, Hemoglobin, White blood cell count at baseline, Previous spontaneous bleeding	(96)
CREDO-Kyoto	Peripheral vascular disease, AF, Malignancy, Prior MI, Severe CKD, Low platelet (<100,000/µl)	(97)
ARC-HBR criterion	Age, Oral anticoagulation, Chronic kidney disease, Anemia, Prior bleeding and transfusion, Thrombocytopenia, Chronic bleeding diatheses, Cirrhosis with portal hypertension, Cancer, Previous ischemic stroke or ICH, Planned major noncardiac surgery after PCI, PCI after recent major surgery or trauma, Long-term oral, NSAID or steroid use	(98)

INR, international normalized ratio; CHF, chronic heart failure; ESRD, end stage renal disease; eGFR, estimated glomerular filtration rate; BMI, body mass index; AF, atrial fibrillation; MI, myocardial infarction; CKD, chronic kidney disease; ICH, intracranial hemorrhage; PCI, percutaneous coronary intervention; NSAID, non steroidal anti-inflammatory drugs; CYP2C9, Cytochrome P2C9.

It has been reported that the occurrence of cancer can increase the risk of bleeding in AF patients (15, 16). In a large cohort of AF patients, the authors found that the risk of ICH increased significantly in patients with prostate cancer (adjusted HR 1.31; 95%CI: 1.06–1.62), and the risk of gastrointestinal (GI) bleeding was higher in patients with colorectal, prostate, ovarian, pancreatic and metastatic cancer as well as myeloma after stopping anticoagulation (99). Similarly, a prospective study also showed that AF patients with cancer had a statistically significant increase in the risk of major bleeding (MB) (adjusted HR 2.02; 95%CI: 1.25–3.27) (84). Surprisingly, Ording et al. found that total cancer status did seem to have no significant association with bleeding risk in AF and cancer patients, whether receiving VKA or NOAC treatment (100). In line with this, a nonsignificant association was observed between bleeding and active cancer as well as remote cancer in the ARISTOTLE Trial (83).

An observational compared the bleeding risk in gastrointestinal cancer (GICA) patients who accepted apixaban or edoxaban anticoagulation, but the cumulative incidence of total bleeding, MB, and clinically relevant non-major bleeding (CRNMB) did not significantly differ between apixaban and rivaroxaban groups (101). However, in patients with GI cancers, especially upper GI cancers, meta-regression analysis revealed that DOACs were associated with higher rates of CRNMB events compared with dalteparin (102). In patients with primary brain tumors or secondary brain metastases, several retrospective studies showed that DOACs do not increased the risk of MB compared with LMWH, but DOACs were associated with lower risk of intracranial hemorrhage (103–106). When choosing LMWH, VKA, or DOACs among cancer patients with cancer-associated thrombosis (CAT), it is recommended to use LMWH instead of DOACs in patients with gastrointestinal or urogenital malignancies, while patients with solid tumors can use DOACs (107, 108).

The evidence of choosing anticoagulants among specific cancer patients with AF is limited. Most studies are observational. A large subgroup analysis of ARISTOPHANES trial retrospectively compared the MB risk of different anticoagulants in AF patients with breast cancer, gastrointestinal cancer, genitourinary cancer, hematologic cancer, and lung cancer. The study showed apixaban had a lower MB risk than warfarin in patients with breast cancer, gastrointestinal cancer, and genitourinary cancer, rivaroxaban and warfarin had a similar MB risk, apixaban had a lower MB risk than rivaroxaban only in patients with breast cancer (109). A small observational study explored the safety of DOACs (apixaban, rivaroxaban, and dabigatran) in patients with breast cancer and AF. The results showed that only rivaroxaban group had 3 cases of major bleeding events and 2 cases of clinically relevant non major bleeding (110). And an observational multicentre study from the AMBER-AF registry showed the MB risk did not entail differences (HR 1.53, 95%CI 0.93–2.53) in patients with AF and breast cancer treated with DOACs or warfarin (111). In a large-scale observational study involving 16,096 patients with AF and active cancer from the United States, stratified analysis showed no statistically significant differences in bleeding risk between warfarin and rivaroxaban as well as dabigatran in breast cancer, colon cancer, lung cancer, and prostate cancer (112). Evidence from a Danish nationwide cohort study suggested a similar 1-year risk of bleeding associated with DOAC compared with VKA among patients with AF and GI cancer (HR 1.12, 95%CI 0.71–1.76) (113). And, another Danish nationwide cohort study also found the 1-year risk of bleeding (hematuria and MB) was comparable in patients with AF and history of urologic cancer (114).

Cancer patients have a higher risk of bleeding after PCI. In a multicenter, observational study of ACS patients recruited for PCI (115), multiple regression analysis showed that the presence of cancer was the strongest independent predictor of bleeding (HR 1.5, 95%CI 1.1–2.1, *p* = 0.015). Data from the Mayo Clinic Cath's lab PCI registry database showed that cancer patients had a 2.8% higher bleeding rate than noncancer patients after PCI over an overall 5-year follow-up (HR 1.73; 95%CI: 1.06–2.83; *p* = 0.03) (88). In addition, Jessica et al. (116) explored some complications after PCI in four major cancers (prostate cancer, lung cancer, breast cancer, and colon cancer) and found that lung cancer, colon cancer, and prostate cancer were associated with an increased risk of bleeding ((OR 1.79, 95%CI 1.56–2.05), (OR 3.65, 95%CI 3.07–4.35), (OR 1.41, 95%CI 1.20–1.65), respectively). In another retrospective study to explore the clinical results for cancer patients after PCI (117), Kanenawa et al. used the PARIS bleeding score (95), PRECISE-DAPT score (96), CREDO-Kyoto risk score (97), and ARC-HBR criterion (98) to evaluate the risk of bleeding in cancer patients after PCI. All scores showed that cancer patients had a higher bleeding risk than noncancer patients (*p* < 0.001). After further exploratory analysis, patients that undergone aggressive cancer therapies such as surgery, radiation, chemotherapy or immunotherapy were associated with a greater risk of major bleeding (117).

## Antithrombotic therapy in cancer patients comorbid with AF and ACS

### Anticoagulation in patients with AF and cancer

Warfarin reduced the risk of stroke by 64% and all-cause death by 26% in patients with nonvalvular AF compared with the placebo group (118). Warfarin is also the only safe anticoagulant in AF patients with rheumatic mitral valve disease or artificial heart valves. However, the use of warfarin requires regular monitoring of the international normalized ratio (INR). The advent of nonvitamin K antagonist oral anticoagulants (NOACs) provides a new choice for AF patients. NOACs have better compliance, effectiveness, and safety, without the need for routine monitoring of coagulation function and INR (119, 120). In four well-known RCTs (RE-LY, ARISTOTLE, ROCKET AF, ENGAGE AF-TIMI), NOACs were not inferior to warfarin in the prevention of stroke or systemic embolism (121–124). In addition, a meta-analysis showed that compared to warfarin, NOACs reduce the risk of stroke by 19% and intracranial hemorrhage (ICH) by 52%, with a similar risk of major bleeding, but increase the risk of gastrointestinal bleeding by 25% (119).

The use of anticoagulants to prevent stroke in AF and cancer patients has been an ongoing challenge. Most cardiologists (63%) regard NOACs as the first choice for anticoagulation (125). A retrospective cohort study of the United States analyzing the use of OACs in patients with cancer and nonvalvular AF found that the use of NOACs increased from 21.8% to 76.2%, whereas the use of warfarin decreased from 78.2% to 23.8% from 2011 to 2016 (126). NOACs appear to be superior to warfarin in AF and cancer. A meta-analysis of three RCTs (ROCKET AF, ARISTOTLE, ENGAGE AF-TIMI 48) showed that NOACs and warfarin had no significant difference in the risk of stroke/SE (RR 0.76; 95%CI: 0.52–1.10), but NOACs significantly reduced the risk of major bleeding (RR 0.79; 95%CI: 0.63–0.99) (127). In line with this, Shah et al. (112) also found that NOACs had a similar risk of ischemic stroke to warfarin in terms of efficacy, and rivaroxaban and dabigatran had a similar risk of severe bleeding to warfarin (HR 1.09 95%CI: 0.79–1.50 *P* = 0.59; HR 0.96 95%CI: 0.72–1.27 *P* = 0.75, respectively) in terms of safety, whereas apixaban had a significantly lower risk of major bleeding than warfarin (HR 0.37 95%CI: 0.17–0.79) (112). However, data from the AMBER-AF registry showed that the incidence of stroke and bleeding did not entail differences in those receiving NOAC and warfarin (adjusted HR of stroke (0.91; 95% CI: 0.42–1.99) or severe bleedings (1.53; 95% CI: 0.93–2.53)) in AF patients with breast cancer (111).

Many antitumor drugs inhibit and compete with the cytochrome P-4503A4 enzyme (CYP3A4) or permeability glycoprotein transporter (P-gp). Thus, the simultaneous use of antitumor drugs and NOACs may cause drug‒drug interactions, which may enhance anticoagulation of NOACs and thereby increase the risk of major bleeding (128–130). Meanwhile, the 2018 European Heart Rhythm Association Practical Guide also indicated that strong inhibitors of CYP3A4 or P-gp should not be used with NOACs in AF patients (128). However, a national retrospective study showed that only 18% of patients with AF and cancer had major bleeding when NOACs were combined with antitumor drugs with inhibitory or competitive effects on CYP3A4 or P-gp activity, which may be explained by the use of low-dose NOACs and cancer-related hypercoagulability (131). The Table 2 showed some common anticancer drugs that can induce or inhibit CYP3A4 or P-gp substrate (128).

**Table 2:** Reported drug—drug interactions between common anticancer drugs and DOACs.

Anticancer drugs	Dabigatran	Apixaban	Edoxaban	Rivaroxaban	Apixaban	Rivaroxaban
	CYP3A4 substrate				P—gp substrate	
	Inducer		Inhibitor		Inducer	Inhibitor
Antimitotic gent						
Paclitaxel	Moderate		—		—	—
Vinblastine	—		—		Strong	—
Docetaxel, Vincristine	Mild		—		—	—
Vinorelbine	Mild		—		—	—
Topoisomerase inhibitors						
Etoposide	—		Mild		—	—
Anthracyclines/Anthrancenediones						
Doxorubicin	—		Mild		Strong	—
Idarubicin	Mild		—		—	—
Alkylating agents	—		—		—	—
Ifosfamide	—		Mild		—	—
Cyclophosphamide	—		Mild		—	—
Tyrosine kinase inhibitors						
Imatinib, Crizotinib	—		Moderate		—	Strong
Nilotinib, Lapatinib	—		Mild		—	Moderate to Strong
Dasatinib	—		Mild		—	—
Vandetanib, Sunitinib	—		—		Strong	—
Hormonal agents						
Abiraterone	—		Moderate		—	Strong
Enzalutamide	Strong		—		—	Strong
Bicalutamide	—		Moderate		—	—
Tamoxifen	—		Mild		—	Strong
Anastrozole	—		Mild		—	—
Immune—modulating agents						
Cyclosporine	—		Moderate		—	Strong to moderate
Dexamethasone	Strong		—		—	—
Tacrolimus	—		Mild		—	Strong to moderate
Prednisone	Moderate		—		—	—
Temsirolimus, Sirolimus	—		Mild		—	—

CYP3A4, cytochrome P-4503A4 enzyme; P-gp, permeability glycoprotein transporter; DOACs, direct oral anticoagulants.

Thrombocytopenia (TP) is relatively common in cancer patients. It has been reported that approximately 10% of cancer patients have less than 10 × 10^9^/L because of cancer and its therapies (132, 133). TP in patients with malignancy is associated with an increased risk of bleeding and ischemic complications (134). In the case of stable TP > 50 × 10^9^/L, the European Hematology Association (EHA) recommends that full-dose NOACs should be superior to warfarin or low-molecular-weight heparin (LMWH) in nonvalvular AF patients with cancer (135). In AF patients with cancer and platelet counts 25–50 × 10^9^/L, a 50% reduction in the LMWH dose may be safe (135, 136). Platelet count <25 × 10^9^/L may have individualized treatment (136, 137). In conclusion, NOACs may be considered the first choice of anticoagulation for AF and cancer patients (138).

### Antithrombotic therapy in patients with cancer and ACS

In the 2017 ESC Guideline on dual antiplatelet therapy (DAPT) in coronary arterial disease, DAPT with aspirin and P2Y12 inhibitor is recommended for 12 months in all ACS patients without high bleeding risk (HBR) but for 6 months in ACS patients with percutaneous coronary intervention (PCI) and HBR and for at least one month in ACS patients with medical therapy alone and HBR (139).

Cancer increases the risk of thrombotic and bleeding events in ACS patients. A retrospective study analyzed 456 patients with acute myocardial infarction (AMI) and active cancer, and only 211 (46.3%) patients used aspirin (140). Additionally, Yusuf et al. (140) analyzed the reasons why cancer patients with AMI did not use aspirin, most of whom had TP (73%). Thirty-nine percent of patients with cancer and thrombocytopenia have been diagnosed with ACS (141). Aspirin can improve the survival of cancer patients with ACS and thrombocytopenia (142, 143). The 7-day survival rate of patients who did not receive aspirin was 6%, while that of patients who received aspirin was 90% (142). The Society for Cardiovascular Angiography and Interventions (SCAI) Expert Consensus Statement recommends that aspirin may be used when platelet counts are >10 × 10^9^/L; DAPT with clopidogrel may be used when platelet counts are 30–50 × 10^9^/L, and prasugrel, ticagrelor and IIB-IIIA inhibitors should not be used in patients with platelet counts <50,000/ml (144). Moreover, if platelet counts are <50 × 10^9^/L, the duration of DAPT may be restricted to 2 weeks after percutaneous coronary angioplasty (PTCA) alone, 4 weeks after bare-metal stents, and 6 months after second- or third-generation drug-eluting stents if optimal stent expansion is confirmed by intravascular ultrasound or optical coherence tomography (144). Aspirin and clopidogrel should be first recommended for ACS patients recently diagnosed with cancer (<12 months) (145, 146). Ticagrelor and prasugrel should not normally be used because of the high risk of bleeding and limited data about their efficacy and safety in patients with active cancer (145). In addition, clopidogrel may occur drug-drug interactions with anticancer drugs through CYP450 (146). To reduce the risk of bleeding, the duration and intensity of DAPT should be minimized (147). In addition, the 2017 ESC Guideline DAPT also recommended that bleeding risk is a major factor affecting the duration of DAPT (139). Thus, the approach of shortening DAPT duration (1–3 months) followed by single antiplatelet therapy is an absorbing option for cancer patients with high bleeding risk (145–148).

### Antithrombotic strategy in cancer patients with ACS and AF

In AF patients with ACS with or without PCI, a combined antithrombotic regimen of antiplatelet and anticoagulant therapy may be needed. Guidelines recommend choosing either dual antithrombotic therapy [DAT: (N)OAC + P2Y12 inhibitor] or triple antithrombotic therapy [TAT: (N)OAC + P2Y12 inhibitor + aspirin] for AF and ACS patients (4, 5, 139, 146, 149). Many RCTs [WOEST (150), ISAR-TRIPLE (151), PIONEER AF-PCI (152), RE-DUAL PCI (153), AUGUSTUS (154), ENTRUST-AF PCI (155)] have confirmed that DAT is noninferior to or even superior to TAT in safety (bleeding risk), while it is similar to TAT in efficacy (stroke, direct thrombosis, cardiovascular events). Similar results were also obtained in four meta-analyses of the above RCTs (156–159). However, these benefits may be accompanied by an increased risk of ischemia (mainly stent thrombosis and recurrent myocardial infarction). Thus, current guidelines probably recommend that AF patients with ACS/PCI be treated with short-term TAT (one week to one month) and then followed by DAT up to 12 months (4, 5, 139, 149). NOACs are recommended for anticoagulants, and clopidogrel is recommended for P2Y12 inhibitors considering that prasugrel and ticagrelor may increase the risk of major bleeding and are the choice of most patients in most experiments (160). In addition, for AF and ACS patients with medical treatment alone, the guidelines recommended DAT treatment for six months followed by NOAC monotherapy as a default strategy (149).

Cancer is regarded as the strongest independent predictor of bleeding (4, 115, 161). Based on the fact that active malignancy is considered one of the main criteria of ARC-HBR (Academic Research Consortium-High Bleeding Risk), 2020 ESC NSTE-ACS Guidelines recommend that AF and cancer patients with ACS should be treated with TAT for a week and then DAT up to 6 months, followed by NOAC alone (4). The 2020 ESC guidelines for the diagnosis and management of AF patients recommend that if the risk of stent thrombosis is low or the risk of bleeding is higher than the risk of stent thrombosis, it is recommended to stop aspirin early (one week) (149). Additionally, 2022 ESC Guidelines on cardio-oncology recommend that NOACs and single antiplatelet therapies (preferably clopidogrel) are the default strategies after short-term triple antithrombotic therapy (up to one week in the hospital) for AF and cancer patients with ACS (147). Given antitumor therapy, complications, more underlying diseases and cancer-related factors such as cancer itself, cancer type, stage, metastasis and activity (161), the choice of the antithrombotic scheme should be individualized, especially the antithrombotic time, in cancer patients with AF and ACS. The risk of blood clots and bleeding in cancer patients should be assessed individually in the decision-making process for anticoagulants.

**Figure 1:**
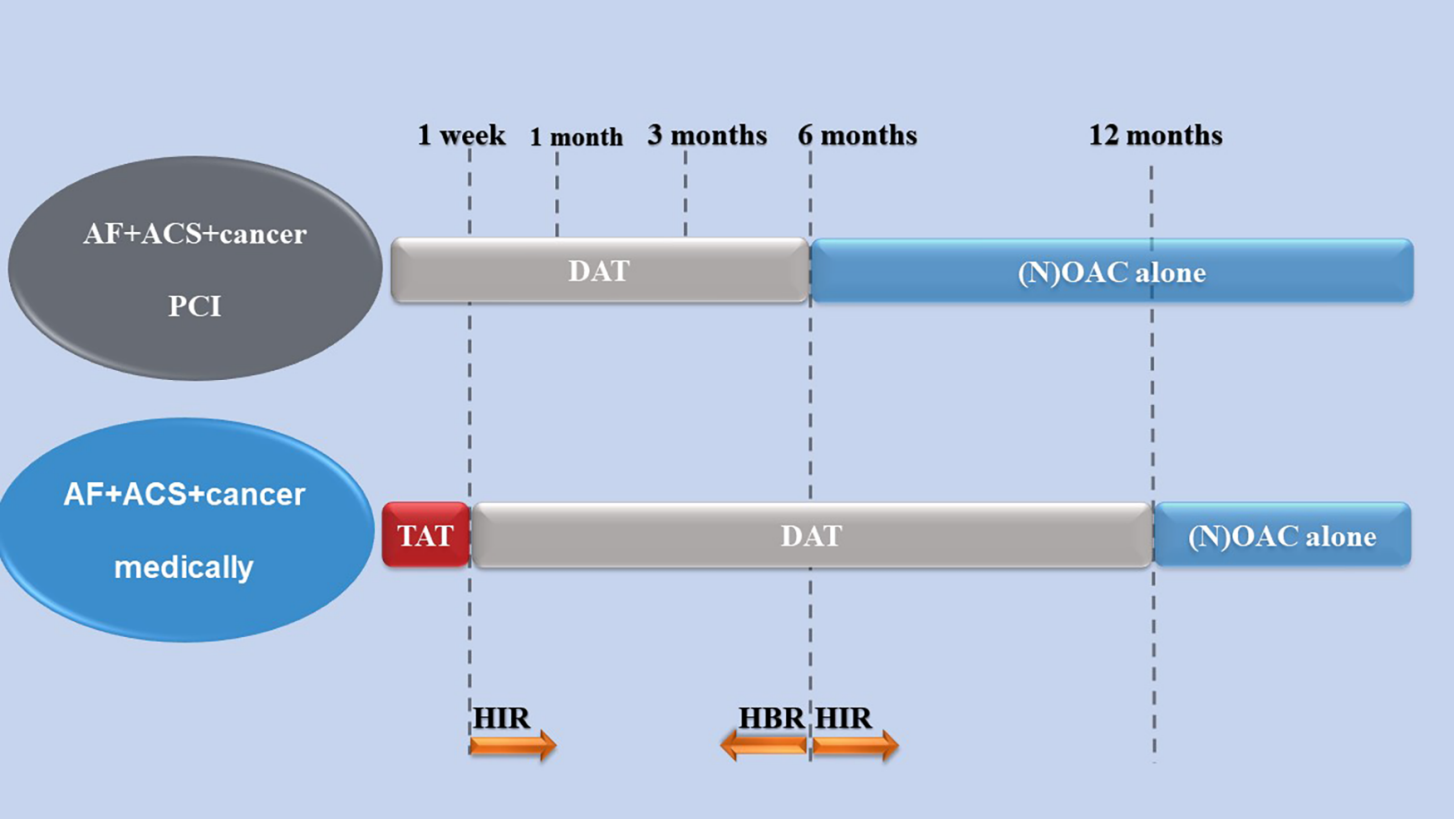
An antithrombotic strategy in patients with cancer comorbid with AF and ACS (4, 147, 149). Arrows indicate the increase or decrease in antithrombotic time. TAT, (N)OAC + P2Y12 inhibitor+aspirin; OAC, oral anticoagulants; NOAC, nonvitamin K oral anticoagulant; DAT, (N)OAC + P2Y12 inhibitor; AF, atrial fibrillation; ACS, acute coronary syndrome; HIR, high ischemic risk; HBR, high bleeding risk.

Currently, the choice of the antithrombotic regimen may be based on the default strategy recommended by the guidelines for AF and ACS patients with cancer (Figure 1), and the best treatment regimen should be determined by individualized and comprehensive assessment of the risk of bleeding and thrombosis (4, 5, 149). The comprehensive evaluation method for bleeding and embolism in AF and ACS patients with cancer is shown in Table 3. Additionally, the dose of anticoagulant may be reduced due to the patient's age, renal function and weight (162).

**Table 3:** A potential method to evaluate the risk of ischemia and bleeding in patients with AF and ACS comorbid with cancer (139, 147, 149, 162).

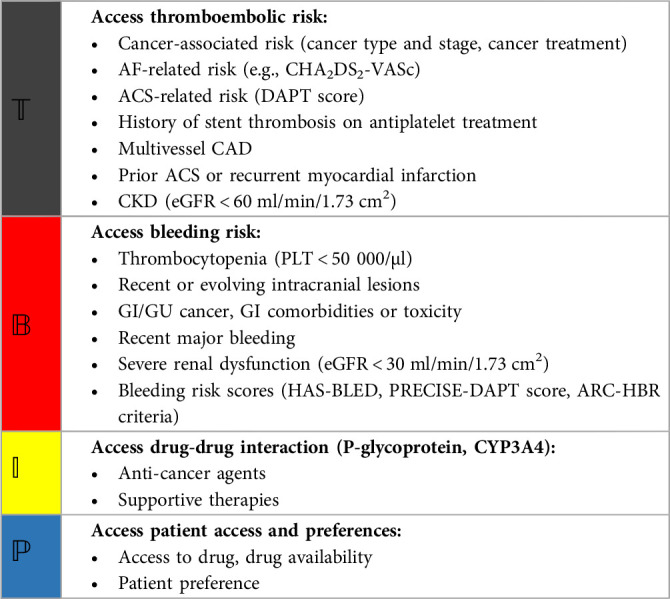

AF, atrial fibrillation; ACS, acute coronary syndrome; CAD, coronary artery disease; CKD, chronic kidney disease; eGFR, estimated glomerular filtration rate; PLT, platelet; GI, gastrointestinal; GU, genitourinary.

### Challenges

**Figure 2:**
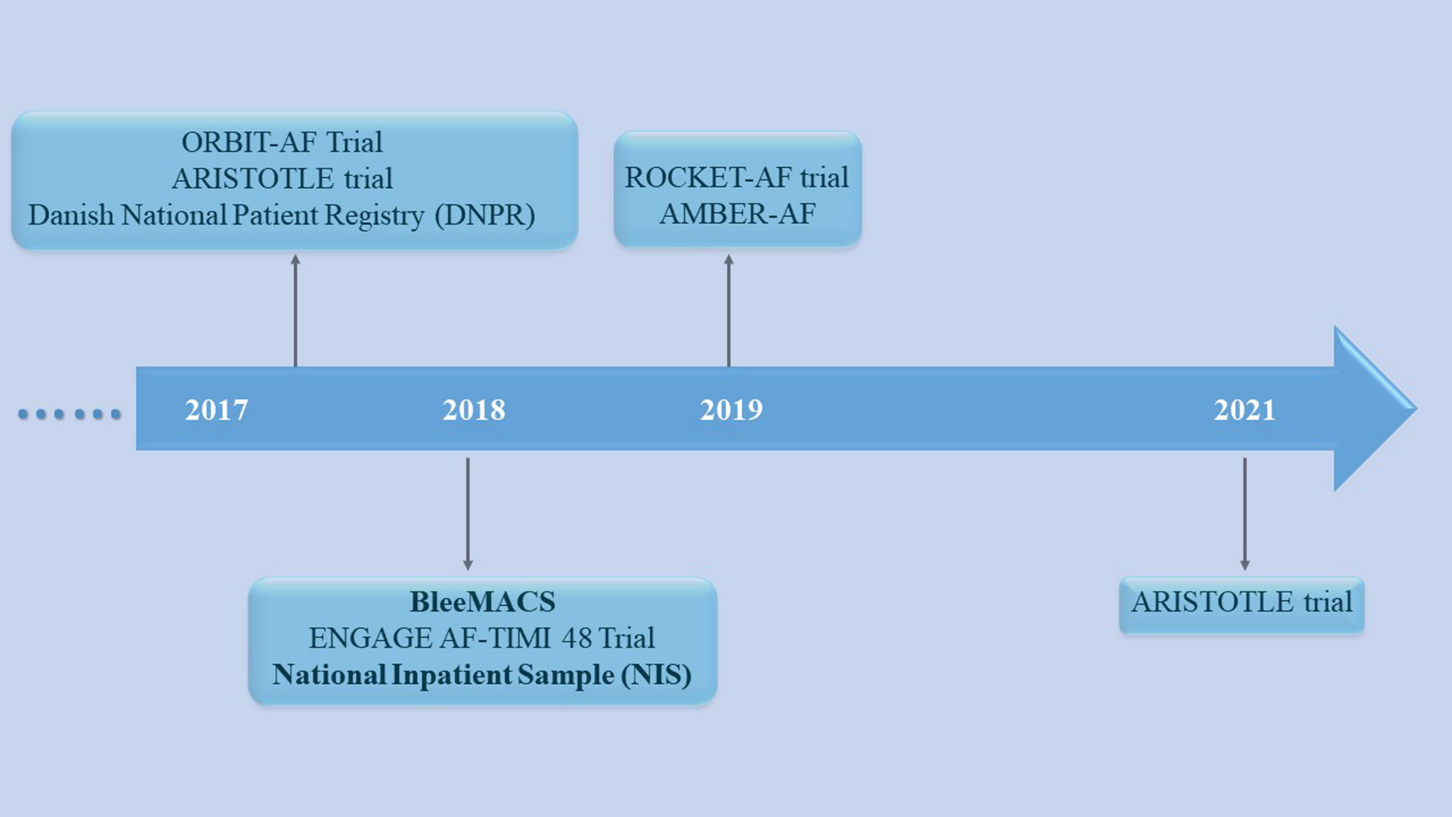
The important mentioned studies for cancer with atrial fibrillation and/or acute coronary syndrome in the review.

Currently, antithrombotic treatment for cancer patients comorbid with CVD, especially AF and ACS, is a challenging issue for clinician, as cancer increases the uncertainty of risk of bleeding and embolism. It is difficult to find the balance between safety and efficacy in the process of antithrombotic therapy. The predictive efficacy of common cardiovascular scores used to assess the risk of bleeding and embolism in cancer patients is relatively low, which makes it difficult for clinical doctors to accurately estimate the risk of bleeding and embolism. In addition, when clinician formulate antithrombotic treatment plans, they need to deal with specific types of cancer, such as gastric cancer. However, most current research on anti-thrombotic therapy for cancer and CVDs only analyzes all types of tumors, which also adds challenges for clinical doctors to make specific decisions. And there is no large-scale RCTs to study anti-thrombotic therapy in cancer comorbid CVD. Most studies are *post hoc* analysis of RCTs (Figure 2). Furthermore, among specific types of cancer, the risk of stent thrombosis in patients with AF undergoing PCI surgery is also difficult to predict, which may increase the risk of recurrent myocardial infarction. In summary, antithrombotic therapy requires individualization in cancer patients with comorbid AF and ACS.

## Conclusion

In cancer patients with comorbid AF and ACS, the selection of a proper antithrombotic strategy is still a challenge for physicians. Most current cardiovascular randomized controlled trials (RCTs) have excluded patients with cancer, resulting in insufficient available evidence in cancer patients with CVD. In addition, scores commonly used to assess bleeding and ischemia risk do not include cancer patients, such as the CHA_2_DS_2_-VASc score, HAS-BLED score, and PRECISE-DAPT score, which may underestimate the risk of bleeding and ischemia in cancer patients. Therefore, individualized antithrombotic therapy is inevitable for AF and ACS patients with cancer. The following problems may need to be solved urgently: (1) develop an embolism and bleeding risk score suitable for patients with CVD and cancer; and (2) implement RCTs to compare the effectiveness of current antithrombotic regimens in patients with cancer and explore the proper antithrombotic strategy in cancer patients comorbid with AF and ACS.
